# Web-Based Video Intervention and Associated Factors for the Uptake of the Catch-Up Human Papillomavirus Vaccination in Japan: Randomized Controlled Trial

**DOI:** 10.2196/67778

**Published:** 2025-08-15

**Authors:** Toshiki Yoshioka, Atsushi Goto, Taichi Mizushima, Yukio Suzuki, Yutaka Ueda, Asami Yagi, Masayuki Sekine, Risa Kudo, Suzanne M Garland, Suresh Kumarasamy, Ida Ismail-Pratt, Katharina Reimer, Etsuko Miyagi, Asia Pacific HPV Coalition

**Affiliations:** 1 Department of Public Health, School of Medicine, Yokohama City University, Fukuura 3-9, Kanazawa-ku, Yokohama, Japan, 81 457872610; 2 Department of Obstetrics and Gynecology, School of Medicine, Yokohama City University, Yokohama, Japan; 3 Department of Gynecology, Kanagawa Cancer Center, Yokohama, Japan; 4 Department of Obstetrics and Gynecology, Graduate School of Medicine, The University of Osaka, Osaka, Japan; 5 Department of Obstetrics and Gynecology, Graduate School of Medical Science, University of the Ryukyus, Okinawa, Japan; 6 Department of Obstetrics and Gynecology, Niigata University Graduate School of Medical and Dental Sciences, Niigata, Japan; 7 Centre for Women’s Infectious Diseases, The Royal Women’s Hospital, Melbourne, Australia; 8 Department of Obstetrics and Gynaecology, University of Melbourne, Melbourne, Australia; 9 Gleneagles Hospital Penang, George Town, Malaysia; 10 The Obstetrics and Gynaecology Centre, Mount Elizabeth Novena Specialist Centre, Singapore, Singapore; 11 Karen Leung Foundation, Hong Kong, China (Hong Kong)

**Keywords:** human papillomavirus, HPV vaccination, behavioral change, education, educational, video, human papilloma virus, papilloma virus, vaccination, Japanese, Asian, woman, randomized controlled trial, unvaccinated, young adult, single-blinded, internet-based, survey, sexually transmitted infection

## Abstract

**Background:**

In Japan, the human papillomavirus (HPV) vaccination rate has dropped to nearly zero since the suspension of proactive government recommendations in 2013. Following the termination of vaccination suspension in 2021 and subsequent proactive vaccination recommendation in 2022, it is crucial to promote catch-up vaccinations for those who missed their initial opportunity.

**Objective:**

This study aims to evaluate the effect of video-based informational intervention and explore factors associated with the uptake of catch-up HPV vaccinations among unvaccinated young adult women in Japan.

**Methods:**

In this randomized, parallel, single-blinded, internet-based trial, we recruited women aged 18‐26 years unvaccinated for HPV through a web-based research panel. The participants were randomly assigned (1:1) to receive either an educational leaflet containing information on the HPV vaccine and a short narrative video (intervention) or the leaflet alone (control). The primary outcome was the difference in proportion between both groups regarding the uptake of the free catch-up vaccinations at the follow-up survey after 3 months. No deviations from the registered protocol occurred during the study.

**Results:**

We enrolled 4065 women in the trial and randomly assigned them to either the intervention (2274 women) or the control (2331 women) group. Of these, we excluded 2595 women (63.8%) who did not respond to the follow-up survey, resulting in 1017 and 993 women in the intervention and control groups, respectively, for the final analysis. At the 3-month follow-up, 11.3% (228/2010) of the participants received at least one catch-up vaccine dose. The intervention and control groups had 10.5% (107/1017) and 12.2% (121/993) uptake, respectively. The difference in proportions between both groups was −1.7% (95% CI −4.5 to 1.2%; *P*=.26), and the adjusted difference was −1.6% (95% CI −4.3 to 1%; *P*=.23). In the subgroup analysis, the intervention group had a lower proportion of catch-up vaccination among sexually experienced women who had previous sexual intercourse experience (difference in proportion −5%, 95% CI −10% to −1%; *P*=.03) and those who had undergone a Pap test within the past 2 years (difference in proportion −11%, 95% CI −20% to −1%; *P*=.03). In addition, in the overall sample, factors positively associated with catch-up vaccination included higher educational background (difference in proportion: 7%, 95% CI 4%-10%; *P*<.001) and having undergone a Pap test within the past 2 years (difference in proportion 5%, 95% CI 0%-9%, *P*=.06).

**Conclusions:**

Our study demonstrated that video-based interventions did not have a substantial impact on the uptake of catch-up HPV vaccinations among young adults. However, our subgroup analyses suggested that the effectiveness of interventions may vary depending on individual characteristics. It is therefore desirable to explore and tailor more effective strategies based on the background and needs of specific populations in real-world settings.

## Introduction

Cervical cancer (CC) is a significant public health issue. Increasing the coverage of the prophylactic human papillomavirus (HPV) vaccine in the target population is essential for CC prevention. Globally, CC is the fourth most common cancer among women, with approximately 662,301 new cases in 2022 [1]. In Japan, approximately 10,000 women are diagnosed with CC, and 3000 women die from the disease annually [2]. In addition, it is the third most frequent cancer among women aged 15‐44 years [1]. Persistent infection with carcinogenic HPV is the cause of almost all CCs [3,4]. HPV vaccination substantially reduces the CC risk at the population level and plays a crucial role in eliminating CC [5-7].

Due to Japan’s governmental suspension of HPV vaccine recommendations between 2013 and 2022, many women in Japan missed the opportunity to receive HPV vaccinations. HPV vaccinations were introduced in fiscal year (FY) 2009 and were formally included in the National Immunization Program (NIP) in April 2013 for girls aged 12‐16 years. However, just two months later, in June 2013, the Ministry of Health, Labour and Welfare (MHLW) suspended its active recommendation for the vaccine in response to a rise in media reports of young girls experiencing chronic pain, numbness, and mobility issues after vaccination. Although the vaccine remained part of the NIP, this suspension of active recommendation led to a dramatic decline in coverage for the target population, girls aged 12‐16 years dropping to nearly zero [8], a situation referred to as a “vaccine crisis” [9,10]. Although the Vaccine Adverse Reactions Review Committee later found no robust evidence linking the vaccine to these events, the suspension was maintained pending further investigation. As accumulating evidence from both domestic and international studies showed that severe adverse events were no more frequent than expected [11], confidence in the vaccine’s safety gradually returned. In November 2021, acknowledging the robust scientific evidence supporting the vaccine’s safety and the rising incidence of CC, the MHLW decided to resume active recommendations for HPV vaccination for girls aged 12‐16 years, effective from April 2022. It is essential to promote vaccination for women who missed the opportunity during this “temporary suspension” period. Consequently, a catch-up vaccination programme was started for women born between FY 1997 and FY 2007 who missed the recommended routine immunization [12]. A simulation study indicated that the catch-up immunization coverage among unvaccinated women needed to reach 90% by FY2022 to lower the risk to a similar level or below that of women born between 1994 and 1999 [13]. However, the HPV catch-up vaccination rate among unvaccinated women remained low (12%‐21%) at the end of FY 2022 [14].

Developing effective communication strategies to enhance voluntary vaccine uptake after this period of unacceptance is critical to promote vaccination behavior among the target generation of the catch-up vaccination. The factors influencing vaccine hesitancy are complex and depend on context [15]. There have been numerous intervention trials targeting parents of the vaccination generation, and our group has accumulated evidence in this field [16,17]; however, fewer interventions target young adults. Current evidence on the effects of the interventions on young adults indicates that multicomponent and dialogue-based interventions, such as the involvement of religious or traditional leaders, social mobilization, social media, and mass media are the most effective strategies to address vaccine hesitancy [18]. Reportedly, video-based informational interventions have positive effects on promoting voluntary vaccination, although they do not necessarily translate into positive behavioral outcomes of increased vaccination rates [19-21]. However, no study has targeted the generation Z (Gen Z) born between the late 1990s and the early 2000s [22]. Gen Zs are genuine digital natives, having been exposed to the internet, social networks, and mobile devices from a very young age, and digital media has a greater influence on their attitudes than the previous generation [23]. Therefore, the effectiveness of video interventions on Gen Z may differ from that of the previous generations of young adults. Furthermore, the factors associated with catch-up HPV vaccine uptake in this generation may also differ from those reported in earlier studies, many of which focused on parental decision-making for vaccinating their children, rather than individuals making vaccination decisions for themselves.

This randomized controlled trial (RCT) aims to address this knowledge gap by assessing the effect of video-based informational intervention on the uptake of catch-up HPV vaccinations among unvaccinated Gen Z women living during the period of suspension of proactive government recommendations of HPV vaccination. In addition, we explored factors associated with catch-up vaccine uptake to better understand the determinants of vaccination behavior in this population.

## Methods

### Trial Design and Setting

We conducted this randomized, controlled, parallel, single-blinded, purely web-based trial targeting women aged 18‐26 years eligible for catch-up HPV vaccinations. This trial was conducted from August 28, 2023, to September 6, 2023, as a pre-survey, and from December 7, 2023, to December 20, 2023, as a follow-up survey. Participants were randomly assigned in a 1:1 proportion to a control group (those who solely read a leaflet on free catch-up vaccinations) and an intervention group (those who additionally watched a brief interventional video), with the primary outcome of accepting HPV vaccine assessed at 3 months.

### Ethical Considerations

The Yokohama City University Ethics Committee approved this study (F230706002). All procedures were conducted in accordance with the study protocol, and no deviations from the protocol occurred. Informed consent was obtained electronically from all participants prior to enrollment. Participants were provided with a detailed explanation document outlining the study purpose, procedures, potential risks and benefits, confidentiality assurances, and their right to withdraw at any time without penalty. All data were collected anonymously through a secure web-based platform. No personally identifiable information was obtained, and responses were stored in a deidentified format to ensure confidentiality and privacy. Participants were recruited through commercial web-based research panels and were incentivized through a point-based reward system managed by each panel provider.

### Participants and Trial Procedures

Women aged 18‐26 years who missed the opportunity to receive the HPV vaccine during the NIP (ages 12‐16) and had not been vaccinated for HPV at the time of this study were recruited from the registered members of the Nippon Telegraph and Telephone (NTT) Com Online Marketing Solutions Corporation (Tokyo, Japan) research panel. Participants were incentivized through a point-based reward system managed by each survey panel provider. The amount and form of incentives varied across platforms and individuals, and the points could be exchanged for gift cards or other rewards. Inclusion criteria included the ability to use a computer and access the internet. We excluded women whose HPV vaccination status was unknown. This panel is used for customer satisfaction evaluations, promotional strategies, and academic studies, and about 53,000 individuals in Japan have enrolled in this research panel [24]. Before participation, all eligible individuals were provided with a detailed explanation document and informed consent form. The document outlined the purpose and significance of the study, procedures and duration, voluntary participation and the right to withdraw without penalty, data handling and confidentiality, potential risks and benefits, and contact information for inquiries. We randomly assigned participants using the year of birth in three-year increments (FY 1997–1999, FY 2000–2002, and FY 2003–2005) as a stratifying factor. We stratified by year of birth because individuals from different birth cohorts may have been exposed to varying social and technological environments during adolescence, which could influence their attitudes and behaviors regarding HPV vaccination. In particular, younger cohorts are more likely to be digital natives and to rely on social media for health information. Furthermore, neurodevelopmental differences between younger and older participants—such as variations in cognitive and emotional maturity—may also affect health-related decision-making [25]. The NTT Com Online Marketing Solutions Corporation data center generated random numbers using computers and performed simple randomization within each stratum. The allocation information was not disclosed to the participants (single-blinded). After responding to the initial web-based survey, participants were asked to view a leaflet issued by the MHLW regarding free catch-up HPV vaccination [26]. The leaflet provided information on the availability of the HPV vaccine for women born between FY 1997 and FY 2006 who missed the opportunity to get vaccinated. The leaflet explained what the HPV vaccine is, the types of HPV it protects against, and the importance of getting vaccinated to prevent CC. It also included details on the potential side effects of the vaccine and emphasized the importance of regular CC screenings for women >20 years old. The leaflet was developed by the MHLW with a general audience in mind and was written using plain language to ensure accessibility for individuals with varying levels of health literacy. This pre-survey consisted of 10 items and gathered comprehensive data, including their age, year of birth, residential area (by prefecture), household and personal income, highest educational attainment, reasons for not receiving the HPV vaccine, whether they had undergone a cervical cytology screening in the past 2 years, sexual intercourse experience, intention to receive the HPV vaccine within the next 3 months, and their communicative and critical health literacy (CCHL) scores [27]. It also assessed their awareness with specific questions such as:

- Were you aware that you are eligible for the free vaccination against HPV since 2022?
- Were you aware that the infection of the cervix with HPV can cause CC?
- Were you aware that CC screenings are recommended every 2 years for individuals ≥20 years?

After viewing the leaflet, those in the intervention group were required to watch a brief, approximately 50 second intervention video featuring two female medical students discussing the catch-up HPV vaccination and its safety [28]. The video was intentionally designed as a peer-to-peer dialogue to enhance engagement and relatability among the target population. In the video, a student expresses her mother’s concerns about potential side effects, while the other explains that millions of women around the world have received the vaccine and that she herself was vaccinated without any issues. This conversational format aimed to encourage viewers to feel included in the discussion rather than simply being presented with facts, thereby incorporating principles of dialogue-based intervention. The tone of the video was both informative and engaging, intended not only to convey key facts but also to evoke a subtle emotional response by fostering empathy and a sense of inclusion—potentially making it more persuasive than an informational leaflet alone.

After 3 months, a follow-up survey consisting of 11 items was administered web-based to evaluate outcomes. This survey obtained information on whether participants had received catch-up vaccinations, the type of vaccine administered (bivalent, quadrivalent, or nonavalent), the subjective understandability of the leaflet, CCHL scores, and their awareness of HPV and HPV-related cancers, using questions such as:

- Were you aware that HPV can cause cancers in men as well (for example, in the throat, penis, and anus)?
- Were you aware that the HPV vaccine is administered to young boys and men in some countries?
- Were you aware that in Japan and abroad, there has been a significant reduction in pre-cancerous lesions among young women who were vaccinated with the HPV vaccine?

For participants who had not received catch-up vaccinations, we inquired about their plans regarding future vaccination. In addition, among those who did not plan to receive catch-up vaccinations, we investigated the reasons behind their decision. Participants in the control group were given access to the brief video before responding to the follow-up survey to ensure equal opportunity. A complete list of the pre- and post-survey items is provided in Multimedia Appendix 1.

### Outcome Measures

The primary outcome was the difference between the two groups regarding the uptake of free catch-up vaccinations at the follow-up survey. The secondary outcomes were as follows: the difference between the two groups at the follow-up survey regarding the uptake of the nonavalent HPV vaccine, awareness about HPV or HPV-related cancer, the change in CCHL score, and the subjective understandability of the leaflet. In addition, we explored individual-level factors associated with HPV vaccine uptake in order to better understand characteristics influencing vaccination behavior.

### Independent Variables

The continuous independent variables were age (years) and both household and personal income (Japanese yen, entered as numeric values); household income was also converted to quintiles (Q1-Q5) for modeling. Categorical variables were defined as follows: year of birth (1997‐1999, 2000‐2002, and 2003‐2005); residential region (Hokkaido, Tohoku, Kanto, Chubu, Kinki, Chugoku, Shikoku, and Kyushu); highest educational attainment (higher education=graduate school, university or junior college; lower education=all other levels); sexual intercourse experience (self-reported; response options: yes, no, or unsure); cervical cytology screening in the past two years (self-reported; yes, no, or unsure); HPV-related knowledge score from the pre-survey (0, 1, 2, or 3 correct answers); 3 post-survey HPV knowledge items (each coded as correct, incorrect, or unsure); CCHL dichotomized at the sample median (high vs low); and perceived understandability of the leaflet (understandable=strongly or somewhat agree and not understandable=other responses).

### Statistical Analysis

We hypothesized that the additional viewing of the video would show superiority to reading only the leaflet. Considering the potential intervention effect of participating in the study, including viewing the leaflet, we set the vaccination rate for the control group at 10% over 3 months. We estimated that the 3-month vaccination rate would be around 3%‐5% without any intervention, based on a recent study [14]. However, given that all participants were exposed to the MHLW leaflet, which may have had a positive effect on awareness and intention, we assumed a 10% vaccination rate in the control group. We defined the clinically meaningful minimum difference in vaccination rates between both groups as 5%, setting the vaccination rate in the video intervention group at 15%. Using the Fisher exact method with a 2-sided significance level of 5% and a power of 80%, we calculated that 721 participants were needed per group. However, anticipating dropouts, we aimed for 2000 participants, with 1000 in each group. We included those who registered for the study and responded to all survey questions after randomization. Due to the nature of the web-based survey, those who did not respond to the follow-up survey were considered as not willing to participate. To ensure data quality, we excluded respondents whose self-reported age and year of birth were inconsistent between the pre- and post-surveys.

The difference in proportions between the two groups was estimated using the Fisher exact test. Wald CI was used to calculate the CIs for the difference in proportions between the two groups. R version 4.3 (The R Foundation for Statistical Computing) was used for statistical analysis and report generation. The significance level for tests was set at a 2-sided 5%.

For the primary outcome, we calculated the proportion and difference between the intervention and control groups regarding taking up the vaccine during the study period. We also conducted a modified least squares regression with robust standard errors to adjust for potential confounding factors [29]. HPV vaccine uptake (yes or no) was entered as the dependent variable. Independent variables were prespecified in the study protocol on theoretical relevance and forced into the model: year of birth, sexual experience, residential area, highest educational attainment, household income, main reason for never receiving the HPV vaccine, cervical cytology screening within the past 2 years, intention to receive the HPV vaccine within the next 3 months, and baseline CCHL score. Subgroup analyses were conducted to evaluate the heterogeneity of the intervention effect. The subgroups included the year group, sexual intercourse experience, residential region, educational background, household income, the reasons for not being vaccinated, knowledge of HPV, Pap test history in the last 2 years, intention to be vaccinated in the following 3 months, and CCHL score in the pre-survey. This involved calculating the difference in the primary outcome between the two groups within each stratum, along with their 95% CIs. These subgroup analyses allowed us to explore how the effect of the intervention varied by individual characteristics, which may help inform the development of more targeted and effective communication strategies for different demographic or behavioral groups. Multiple regression analyses were applied to the control group, the intervention group, and the entire study participants while adjusting for potential confounders to explore the effects of various factors on the catch-up vaccination behavior. Robust variance estimation was used in this analysis. The factors included the year group, educational background, household income, intercourse experience, knowledge of HPV, Pap test history in the last 2 years, and CCHL score in the pre-survey (Table S1 in Multimedia Appendix 1).

We conducted analyses for the secondary outcomes as follows: We calculated the proportion of participants in the 2 groups who received the nonavalent HPV vaccine with the same method as the primary outcome. The crude and adjusted differences in the proportions of participants between the two groups with awareness about HPV or HPV-related cancer in the follow-up survey were calculated with their 95% CIs. We also calculated the crude and adjusted differences of the CCHL scores between the follow-up survey and pre-survey, along with the average differences between the two groups and their 95% CIs. The adjusted differences were calculated using multiple regression analysis. We applied similar potential confounders used in the primary outcome of these analyses. Furthermore, to explore the characteristics associated with the subjective understandability of the leaflet, multiple regression analyses were applied to the entire study population while adjusting for similar potential confounders used in the analyses of factors affecting catch-up vaccination behavior (see Table S1 in Multimedia Appendix 1). Robust variance estimation was used in this analysis.

Finally, among women who had not received a catch-up vaccination at the time of the follow-up survey and indicated that they did not plan to receive the HPV vaccine in the future, we descriptively summarized their reasons for this decision. The categorization for this summary was based on classifications from previous studies on vaccine hesitancy [30]. This qualitative assessment helped characterize those who remained vaccine-hesitant despite the intervention, providing further insight into potential barriers and informing future approaches to address specific concerns within this group.

## Results

### Trial Population

**Figure 1.**
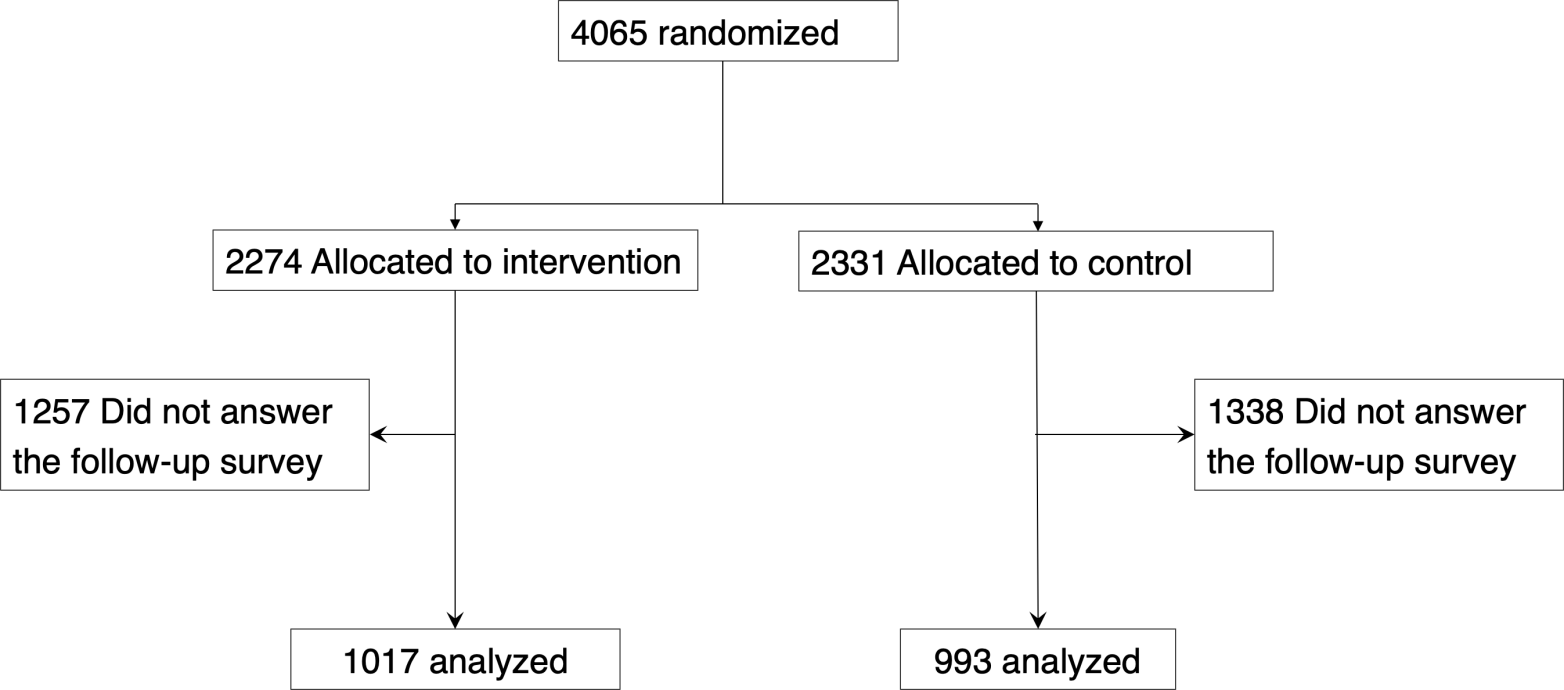
Flow diagram of the randomization.

We enrolled 4065 women in the trial from August 28, 2023, to September 6, 2023, and randomly assigned them to either the intervention group (n=2274 women) or the control group (n=2331 women). In the intervention group, 1257 women did not answer the follow-up survey, leaving 1017 women to be analyzed for the primary outcome. In the control group, 1338 women did not answer the follow-up survey, leaving 993 women to be analyzed for the primary outcome. Therefore, 2595 women (63.8%) were excluded from the study population because they did not answer the follow-up survey, which left 1017 women in the intervention group and 993 women in the control group for the final analysis (see Figure 1). The median age was 22 years (IQR 20‐24 y) for both groups, and other baseline characteristics were similar (see Table 1). The median time to complete the pre-survey was 2 minutes and 20 seconds, and the post-survey took a median of 2 minutes and 5 seconds.

**Table 1.** Characteristics of the participants at baseline.

Characteristic	Control (N=993)	Intervention (N=1017)	*P* value^a^
Age (years), median (IQR)	22 (20-24)	22 (20-24)	.76
Region, n (%)			—^b^
Hokkaido	36 (3.6)	38 (3.7)	
Tohoku	70 (7)	84 (8.3)	
Kanto	356 (36)	360 (35)	
Chubu	165 (17)	172 (17)	
Kinki	194 (20)	221 (22)	
Chugoku	57 (5.7)	53 (5.2)	
Shikoku	19 (1.9)	17 (1.7)	
Kyushu	96 (9.7)	72 (7.1)	
Household income (×10,000 Yen^c^), median (IQR)	400 (200-700)	400 (200-600)	.86
Educational background, n (%)			.64
High (junior college level or above)	368 (37)	366 (36)	
Low	625 (63)	651 (64)	
History of sexual intercourse, n (%)			.05
Yes	430 (43)	480 (47)	
No	424 (43)	427 (42)	
Unsure	139 (14)	110 (11)	
Intention to receive HPV^d^ vaccine in the next 3 months, n (%)			.27
Yes	89 (9)	102 (10)	
No	631 (64)	666 (65)	
Unsure	273 (27)	249 (24)	
CCHL^e^ score in the pre-survey, median (IQR)	3.20 (3.00-3.80)	3.20 (3.00-3.80)	.99

a Age and communicative and critical health literacy (CCHL) score—Welch 2‑sample *t* test; all other variables—Fisher exact test.

b Not available.

c Study-period average exchange rate US $1 = ¥148 (Bank of Japan historical rate).

d HPV: human papilloma virus.

e CCHL: communicative and critical health literacy.

### Primary Outcome

Regarding the primary outcome, overall, 11.3% (228/2010) of the participants had received at least one catch-up vaccine dose by the 3-month follow-up survey (see Table 2). This proportion was 10.5% (107/1017) and 12.2% (121/993) in the intervention and control groups, respectively. The difference in proportions between the groups and its 95% CI was −1.7% (−4.5 to 1.2). After adjusting for potential confounding factors, the adjusted difference in proportion was −1.6% (−4.3 to 1.0).

**Table 2.** The results of the primary and secondary outcomes.

Outcome	Overall (n=2010), n (%)	Control (n=993), n (%)	Intervention (n=1017), n (%)	Difference in proportion^a^ (95% CI)^b^	Mean difference (95% CI)^c^	*P* value ^c,d^	Adjusted difference in proportion (95% CI)^e^	Adjusted mean difference (95% CI)^e^
Primary outcome								
Vaccinated (2-, 4- or 9-valent)	228 (11.3)	121 (12.2)	107 (10.5)	−1.7% (−4.5 to 1.2)	—^f^	.26	−1.6% (−4.3 to 1.0)	—
Secondary outcomes								
Vaccinated (9-valent)	68 (3.4)	36 (3.6)	32 (3.1)	−0.4% (−1.2 to 2.1)	—	.62	−0.7% (−2.1 to 0.8)	—
Awareness on the questions								
Awareness on Q1^g^	568 (28.6)	288 (29)	280 (27.5)	−1.5% (−5.5 to 2.6)	—	.49	−2.5% (−6.3 to 1.2)	—
Awareness on Q2^h^	370 (18.4)	182 (18.3)	188 (18.5)	0.2% (−3.3 to 3.6)	—	.95	−0.6% (−3.9 to 2.7)	—
Awareness on Q3^i^	550 (27.4)	278 (28)	272 (26.7)	−1.3% (−5.2 to 2.7)	—	.55	−1.8% (−5.5 to 1.9)	—
Change in CCHL^j^ score^k^	−0.028	−0.041	−0.017	—	0.024 (−0.057 to 0.105)	.56	—	0.083 (−0.070 to 0.086)

a The subtraction of the vaccinated proportion in the control group from the vaccinated proportion in the intervention group.

b Wald interval.

c Fisher exact test.

d Welch *t* test.

e Coefficient of multiple regression analysis, with robust variance estimator.

f Not available.

g “Were you aware that HPV can cause cancers in men as well (eg, in the throat, penis, and anus)?”

h “Were you aware that HPV vaccine is administered also to men in some countries?”

i “Were you aware that in Japan and abroad, there has been a significant reduction in pre-cancerous lesions among young women who have been vaccinated with the HPV vaccine?”

j CCHL: communicative and critical health literacy.

k Mean change.

**Figure 2.**
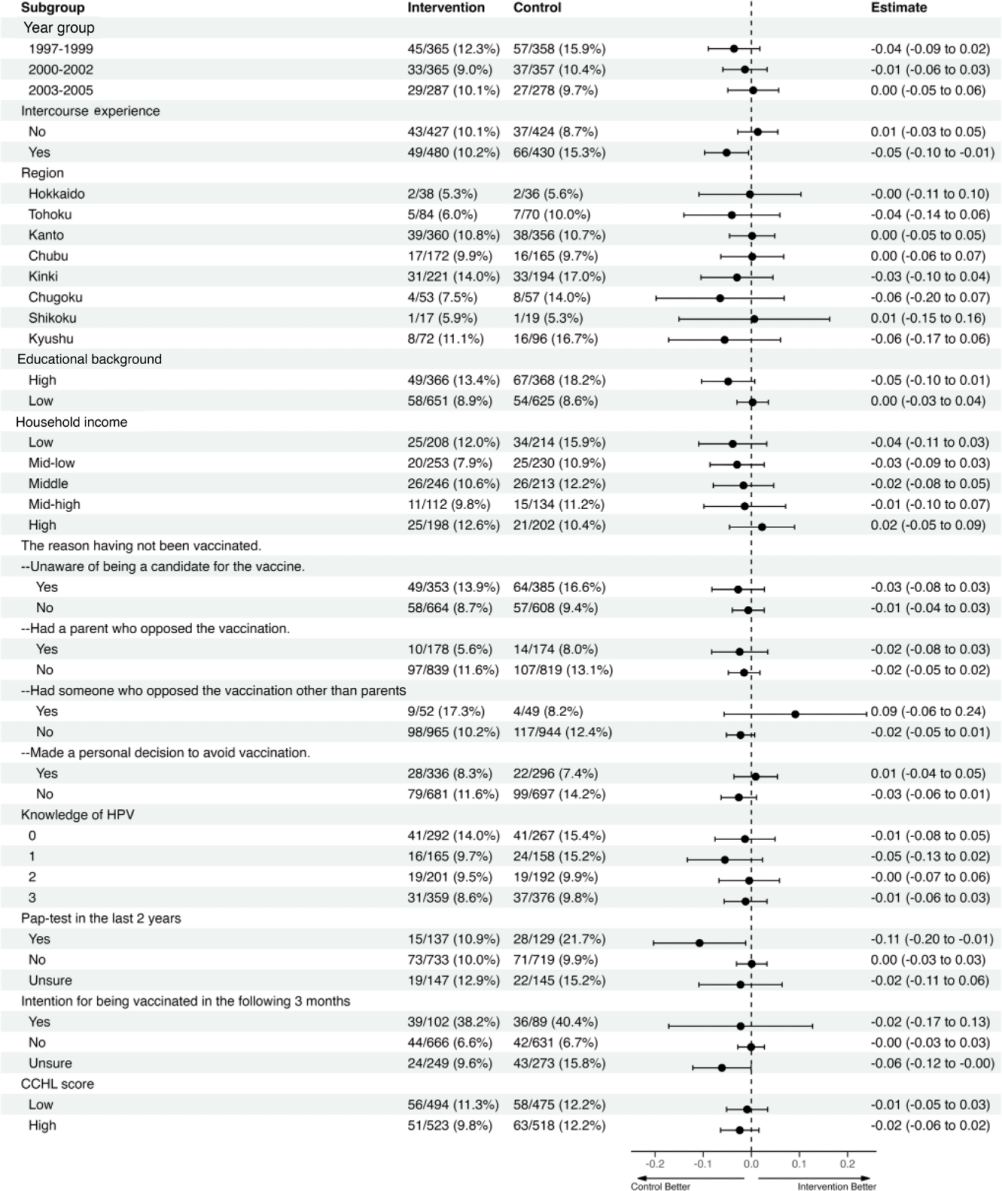
Subgroup analysis. HPV: human papillomavirus; CCHL: communicative and critical health literacy.

Figure 2 shows the subgroup analysis results. The proportion of catch-up vaccination was lower in the intervention group among women who had intercourse experience (difference in proportion: −5%, 95% CI −10% to −1%) and those who had undergone a pap test within the past 2 years (difference in proportion: −11%, 95% CI −20% to −1%). No significant differences were observed in the intervention effect for other factors, including age, residential area, educational background, household income, the reason for not being vaccinated, knowledge of HPV, intention for being vaccinated in the following 3 months, and CCHL score in the pre-survey.

### Factors Associated With Vaccination Behaviors

**Figure 3.**
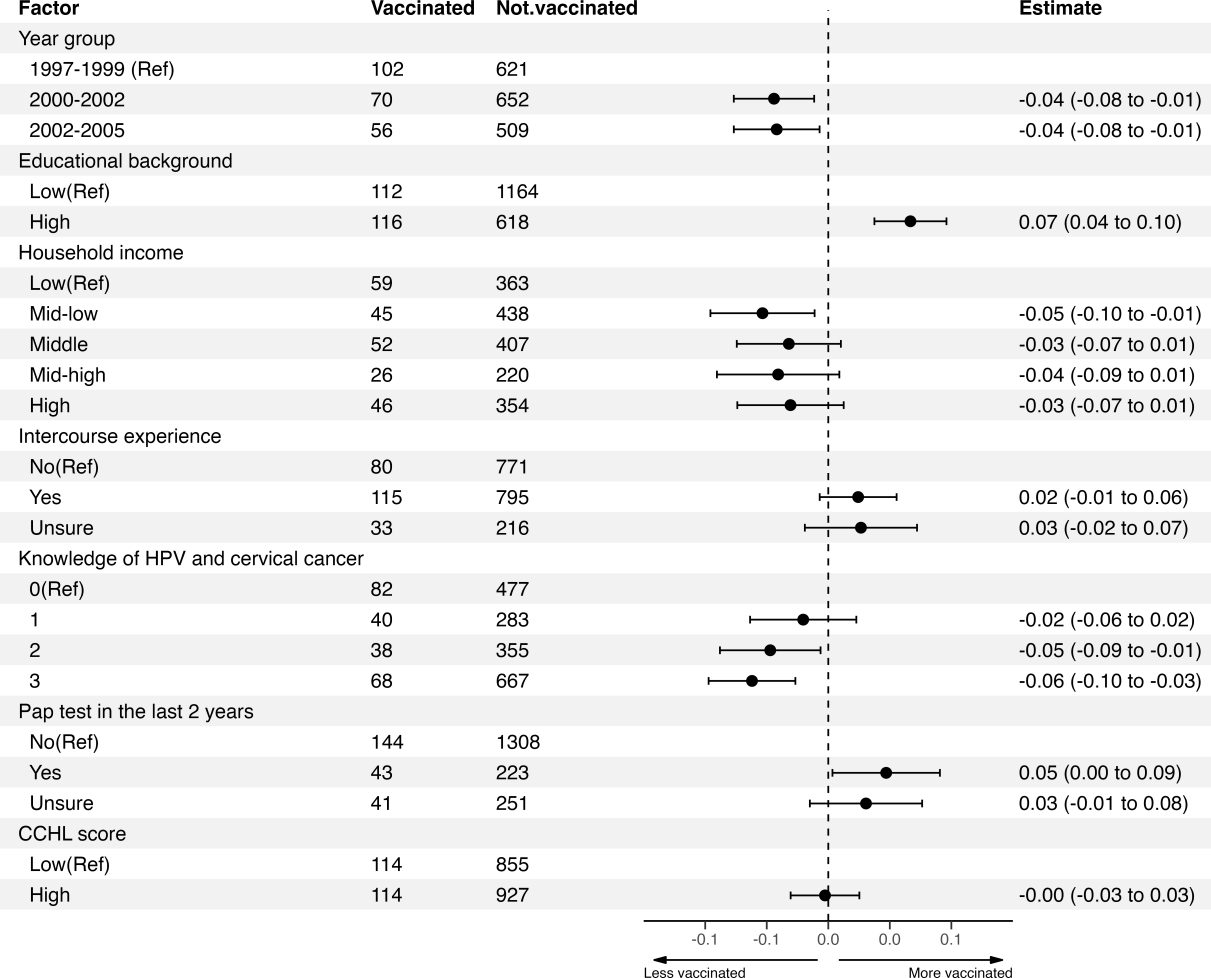
Factors associated with vaccination behaviors in the entire study population. Ref: reference; HPV: human papillomavirus; CCHL: communicative and critical health literacy.

Figure 3 presents the differences in vaccination rates for each factor compared to the reference level after adjusting for confounding factors, in the entire study population. Compared to those born in 1997‐1999 (24‐26 years old), women born in 2000‐2002 (21‐23 years old) and 2003‐2005 (18‐20 years old) had lower vaccination rates at 3 months. Women with lower educational levels had lower vaccination rates compared to those with higher educational levels. In addition, women who had undergone a Pap test within the past 2 years had higher vaccination rates than those who had not. The CCHL score was not associated with vaccination behavior. When we restricted this analysis to the intervention and control groups, we observed similar trends in both groups (Figure S1 and S2 in Multimedia Appendix 1). However, in the intervention group, the confidence intervals for age and having a Pap test in the last 2 years crossed 0, indicating no statistically significant association.

### Secondary Outcomes

Table 2 shows the results of the secondary analysis. The proportion of participants who received the nonavalent HPV vaccine was 3.1% (32/1017) and 3.6% (36/993) in the intervention and control groups, respectively. The difference in proportions between the groups and its 95% CI was −0.4% (−1.2 to 2.1). After adjusting for potential confounding factors, the adjusted difference in proportion was −0.7% (−2.1 to 0.8). As for awareness of HPV and HPV-related cancers, or change in CCHL score between the pre-survey and the follow-up survey, we did not observe significant differences between the intervention and the control groups.

In the analysis of factors associated with the subjective understandability of the leaflet in the entire study population, lower age groups and higher household income were associated with higher subjective understandability (Figure S3 in Multimedia Appendix 1). In addition, there was almost no difference in subjective understandability between those who answered “yes” and those who answered “no” regarding sexual intercourse experience. However, those who answered “unsure” had lower subjective understandability. Similarly, those who answered “unsure” for the Pap test in the last 2 years had lower subjective understandability. Higher subjective understandability was also observed in groups with higher CCHL scores than those with lower CCHL scores.

Figure S4 in Multimedia Appendix 1 shows the reasons given by the 429 individuals who did not receive the catch-up vaccination and indicated that they did not plan to receive the HPV vaccine in the future. These responses were categorized into eight groups based on previous vaccine hesitancy literature: (1) low perceived need or risk (36.1%, 155/429); (2) concerns about vaccine safety (30.3%, 130/429); (3) fear of injections (16.3%, 70/429); (4) information issues, such as not knowing enough about the vaccine (7.5%, 32/429); (5) influence of others (4.4%, 19/429); (6) issues of trust (2.1%, 9/429); (7) doubts about vaccine effectiveness (0.7%, 3/429); and (8) access barriers (0.7%, 3/429). A small proportion selected “other” reasons (1.9%, 8/429).

## Discussion

In this randomized, controlled, internet-based trial, we found that approximately 11% (228/2010) of the participants in the follow-up survey received the catch-up HPV vaccination. However, we were unable to demonstrate an additional benefit of video-based intervention on catch-up vaccination behavior among young adults. Furthermore, women with higher educational levels, older women, or women who had undergone a Pap test within the past 2 years had higher vaccination rates.

The effects of interventions on HPV vaccine uptake among young adults have been inconsistent. There are numerous intervention studies on HPV vaccine uptake. However, most studies target parents of adolescents up to approximately 15 years old or health care professionals, with limited trials focusing on young adults. Furthermore, few interventions demonstrate significant effects on actual vaccine uptake. A RCT performed in the United States reported that a combined peer–expert vaccine decision narrative video doubled the vaccination rate at two months compared to the control group (22% vs 12%) [31]. However, the same RCT found that an expert vaccine decision narrative video alone resulted in lower vaccination rates than the control group (6% vs 12%). Other studies performed in the United States have shown that educational interventions, while increasing vaccination intentions postintervention, do not necessarily translate into actual vaccination behavior [32,33]. In this study, the narrative video featuring female peers did not increase vaccination rates at 3 months. Nevertheless, the fact that 12% of the control group received the vaccine at 3 months suggests that the knowledge-based leaflet itself may have had some intervention effect.

The necessary factor to change the behavior of Gen Z may not be the digital media but rather influencers whom they perceive as trustworthy. Gen Z, known as digital natives, is more influenced by digital media and social media influencers in shaping their attitudes than previous generations [23]. Therefore, we hypothesized that video interventions might have a different impact on this generation compared to earlier cohorts of young adults. However, our results indicate that the narrative video featuring female peers did not increase vaccination rates at 3 months within a Japanese culture. Reportedly, Gen Zs seek influencers who they feel are authentic and are genuinely pursuing interests that they share [34]. From this perspective, our intervention video might have been insufficient to make Gen Zs truly trust or want to follow it, particularly given the large gap of 9 years (2013‐2022) of hesitancy by the government in recommending vaccination. For future interventions, it may be beneficial to recruit influencers who already hold credibility with Gen Z—such as peer educators, university students active on social media, or health-focused content creators. To ensure authenticity, involving young people themselves in the process of selecting or co-creating content with these influencers could enhance relatability and trust, potentially increasing the effectiveness of digital health communication among this demographic.

It is also necessary to remove the barriers between vaccine uptake intention and actual vaccination behavior. According to the theory of planned behavior, intention precedes action [35]. In various previous intervention trials, interventions have increased vaccination intention, but this has not translated into actual vaccination [19-21]. In this trial, we did not measure intention after the intervention; however, individuals need to use the vaccination tickets sent by the municipality to make appointments with health care providers and go to the vaccination site to get vaccinated, and this may act as a barrier. In Japan, for municipally organised voluntary vaccination programs, eligible individuals typically receive a vaccination ticket mailed directly from their local municipal office to their registered residential address. The vaccination ticket includes essential information, such as eligibility, vaccination locations, and instructions for making appointments. In addition, since vaccination tickets are sent from the relevant municipality to the address where the individual is registered, it can be difficult for those whose actual residence differs from their registered address to get vaccinated. It may be important to establish a system that minimizes the barriers between intention and behavior, such as enabling immediate appointment scheduling and vaccination right after the intervention.

The barriers to HPV vaccination vary, and it is necessary to explore the optimal approach to address them. In this study, younger women (18‐20 y and 21‐23 y) had lower vaccination rates compared to older women (24‐26 y), and women with lower educational levels had lower vaccination rates compared to those with higher educational levels. This suggests that educational leaflets may not be suitable for inducing behavioral change in these demographics. The reasons given by women who still expressed reluctance to consider vaccination include “low perceived need or risk” and “concerns about vaccine safety,” which collectively accounted for two-thirds of the responses. Globally, both reasons are among the top reasons for vaccine hesitancy [30]. To overcome these barriers, it is essential to communicate the importance of the vaccine, the risks of not getting vaccinated, and objective facts about side effects in a manner that these women find convincing. Merely providing knowledge is likely insufficient to achieve true understanding and actual behavioral change among these women; therefore, dialogue-based interventions may be necessary [18].

This study had some limitations. First, there was a high dropout rate, which may have threatened the exchangeability between the two groups maintained at the time of random assignment, due to heterogeneity between those who dropped out and those who remained. In web-based intervention trials, nearly 50% of the participants often drop out [36,37], and this study was no exception. Nevertheless, although dropout was substantial, baseline characteristics related to the primary outcome (eg, age distribution, educational background, and CCHL score) remained balanced between the intervention and control groups. Therefore, while the possibility of differential attrition cannot be entirely ruled out, it is considered to be limited, thereby reducing concerns about potential bias resulting from dropout. Second, applying the results from this experimental setting to the real world is challenging. When implementing interventions to promote vaccination, the interventions must be integrated into the actual system. Achieving this at the population level, which includes people who have no intention of getting vaccinated, might be challenging. Furthermore, since the real-world environment can differ significantly from the trial setting, the effects of the intervention might also differ. Third, while we confirmed that the video was played on the participants’ devices in this web-based intervention, we could not verify whether the participants actually watched it. If the actual viewership was low, the intervention effect might be underestimated. Finally, the primary outcome of this study was self-reported through a web-based survey, so we do not have concrete evidence that participants actually received the vaccination.

In conclusion, our study demonstrated that video-based interventions did not have a substantial impact on catch-up HPV vaccinations among young adults, particularly for Gen Z, who are digital natives. However, our analyses suggested that the effectiveness of interventions may vary depending on individual characteristics. It is therefore important to explore and implement more tailored and effective strategies that reflect the backgrounds and needs of specific populations in real-world settings.

## Supplementary material

10.2196/67778 Multimedia Appendix 1 Pre‑survey and post‑survey questionnaires, supplementary table, and figures.

10.2196/67778 Checklist 1 CONSORT-eHEALTH checklist.

## Abbreviations

CC: cervical cancer
CCHL: communicative and critical health literacy
FY: fiscal year
Gen Z: generation Z
HPV: human papillomavirus
MHLW: Ministry of Health, Labor and Welfare
NIP: National Immunization Program
NTT: Nippon Telegraph and Telephone
RCT: randomized controlled trial
