# Supplementary material for: Web-Based Video Intervention and Associated Factors for the Uptake of the Catch-Up Human Papillomavirus Vaccination in Japan: Randomized Controlled Trial
Source: J Med Internet Res. 2025 Aug 15;27:e67778. doi: 10.2196/67778 (PMC12356523; doi:10.2196/67778)

**Pre-survey Questionnaire Items (10 items)**

1. **What is your age?** (Free entry)
2. **Year of birth category:**
   - FY1997–1999
   - FY2000–2002
   - FY2003–2005
3. **HPV-related knowledge:**
   - Were you aware that you are eligible for free HPV vaccination since FY2022?
   - Were you aware that cervical HPV infection causes cervical cancer?
   - Were you aware that cervical cancer screening is recommended every 2 years for individuals ≥20 years?
4. **What is the main reason you have not received the HPV vaccine? (Select one):**
   - I was unaware that I was eligible
   - One of my parents was opposed
   - Someone other than my parents (e.g., friend, teacher) was opposed
   - I decided by myself not to receive it
   - Other (please specify)
5. **Was your decision not to receive the HPV vaccine influenced by past media coverage?**
   - Yes / No / I don't know about past coverage / I don't know or prefer not to answer
6. **Have you undergone cervical cytology (Pap test) in the past 2 years?**
   - Yes / No / I don't know or prefer not to answer
7. **If you were to receive a free HPV vaccine, which of the three types would you choose?**
   - Cervarix (bivalent)
   - Gardasil (quadrivalent)
   - Gardasil 9 (nonavalent)
   - I don't know or prefer not to answer
8. **Have you ever had sexual intercourse?**
   - Yes / No / I don't know or prefer not to answer
9. **Do you plan to get the HPV vaccine within the next 3 months?**
   - Yes / No / I don't know or prefer not to answer
10. **Communicative and Critical Health Literacy (CCHL):**

- I can gather information from various sources (e.g., newspapers, TV, internet).
- I can select the information I need from the available information.
- I can understand and explain the information to others.
- I can judge how trustworthy the information is.
- I can make health-related decisions or plans based on the information.
  *Response options for each item: Strongly disagree / Disagree / Neutral / Agree / Strongly agree*

**Post-survey Questionnaire Items (11 items)**

1. **What is your age?** (Free entry)
2. **Year of birth category:**
   - FY1997–1999
   - FY2000–2002
   - FY2003–2005
3. **HPV vaccination uptake in the past 3 months:**
   - 0 times / 1 time / 2 times
4. **Do you plan to receive the HPV vaccine in the future?**
   - Yes
   - No (if no, please specify reason)
   - I don’t know or prefer not to answer
5. **Which type of HPV vaccine did you receive?**
   - Cervarix / Gardasil / Gardasil 9 / I don’t know
6. **Was the MHLW leaflet easy to understand?**
   - Strongly disagree / Disagree / Neutral / Agree / Strongly agree
7. **Is your registered address the same as your current residence?**
   - Yes / No / I don’t know or prefer not to answer
8. **Did you receive a vaccination ticket from your municipality?**
   - Yes / No / I don’t know or prefer not to answer
9. **Additional HPV-related knowledge:**
   - Were you aware that HPV can cause cancers in men (e.g., throat, penis, anus)?
   - Were you aware that the HPV vaccine is administered to young boys and men in some countries?
   - Were you aware that HPV-related precancerous lesions have significantly decreased in vaccinated women both in Japan and internationally?
     *Response options: Yes / No*
10. **Communicative and Critical Health Literacy (CCHL):**
    (Same 5 items and response options as in the pre-survey)
11. **Have social media videos ever influenced your everyday behavior?**

- Yes / No / I don't know or prefer not to answer

Table S1.

List of adjusted potential confounders for each factor to explore the effects on the catch-up vaccination behavior.

| **Factor** | **Adjusted Confounders** |
| --- | --- |
| The year group | None |
| Educational background | The year group |
| Household income | The year group, educational background |
| Intercourse experience | The year group, educational background, household income |
| Pap-test history in the last two years | The year group, educational background, household income, intercourse experience |
| CCHL score in the pre-survey | The year group, educational background, household income |

Figure S1.

Factors associated with vaccination behaviors in the intervention group


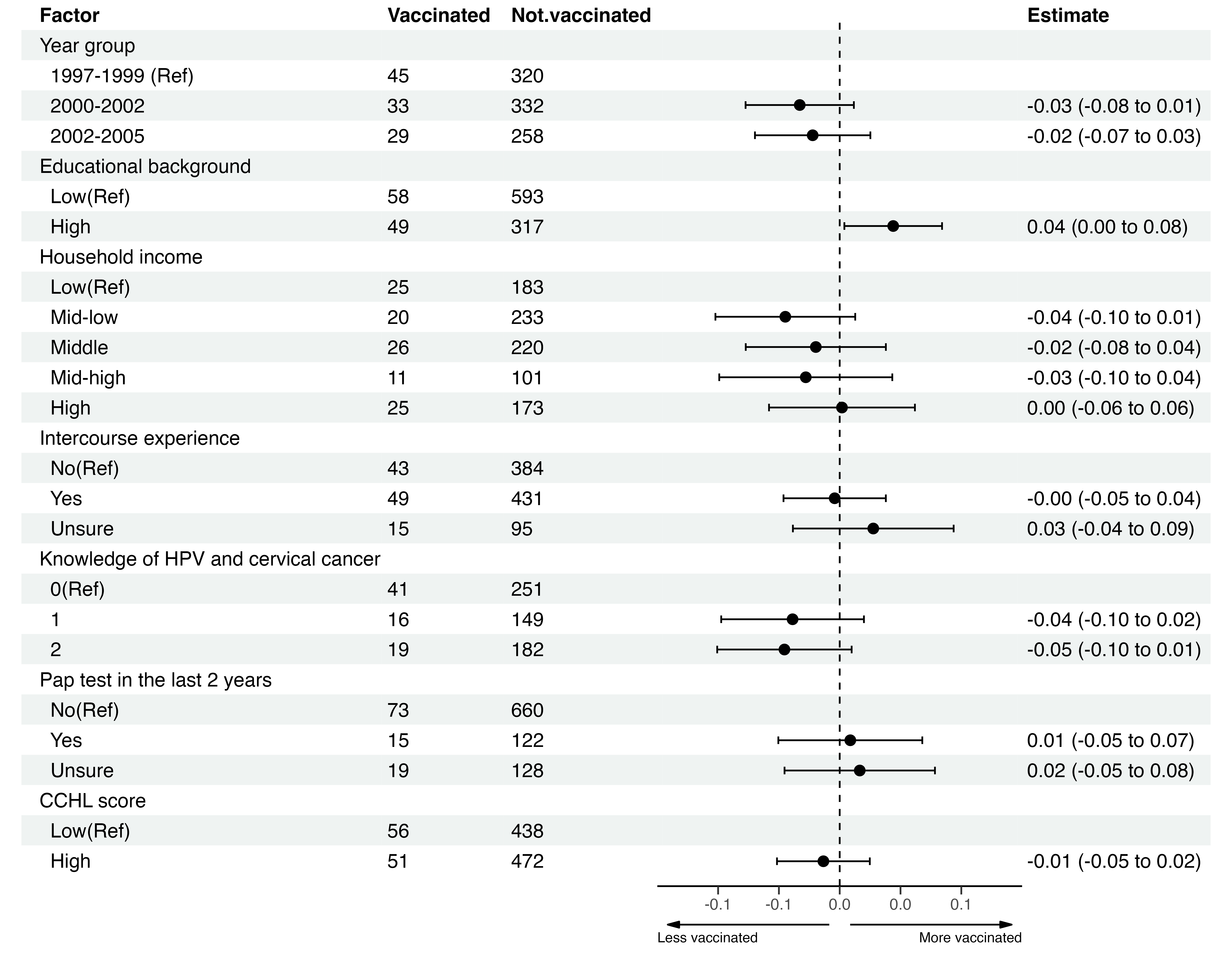


Figure S2

Factors associated with vaccination behaviors in the control group.

**
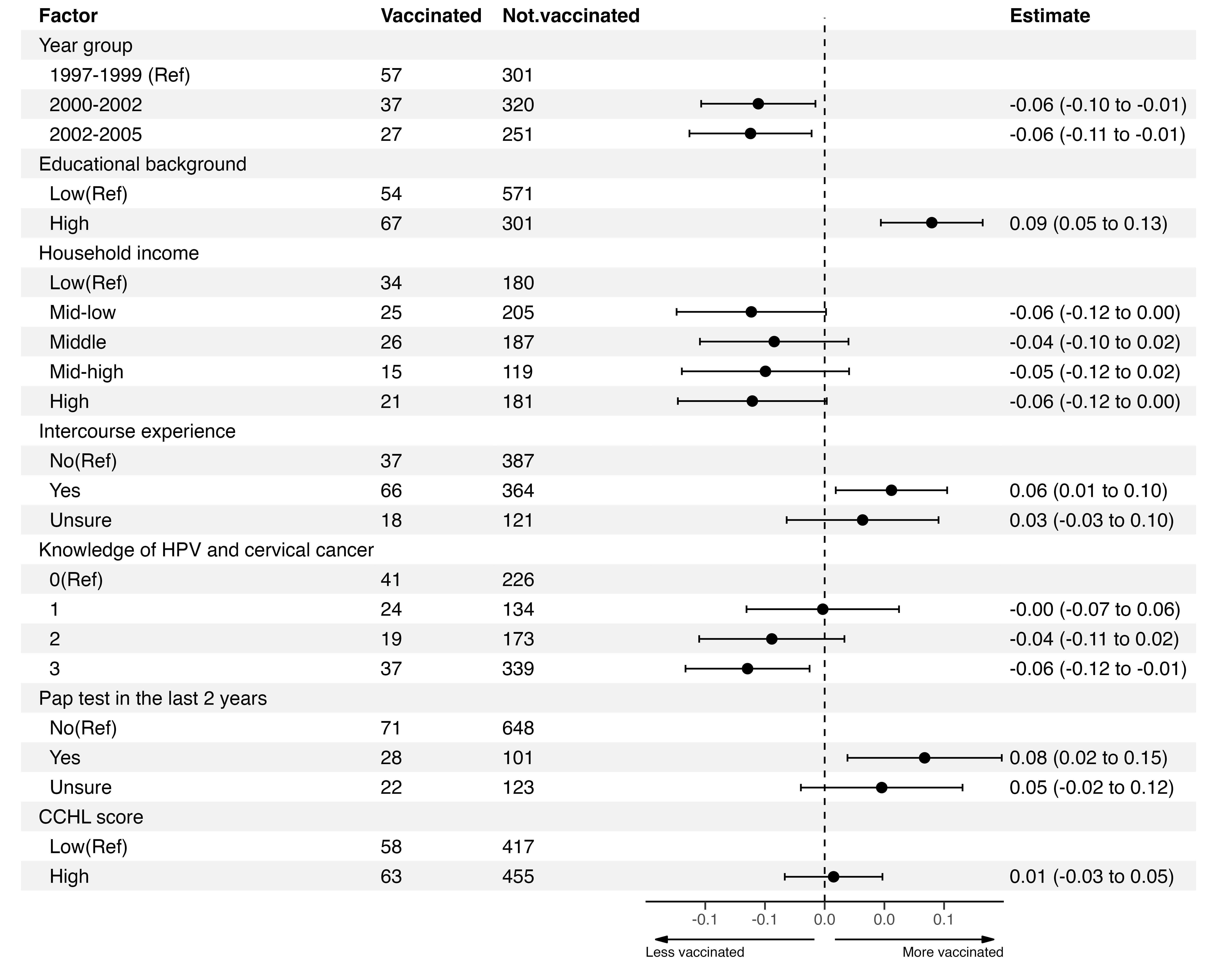
**

Figure S3

Factors associated with the subjective understandability of the leaflet in the entire study population.


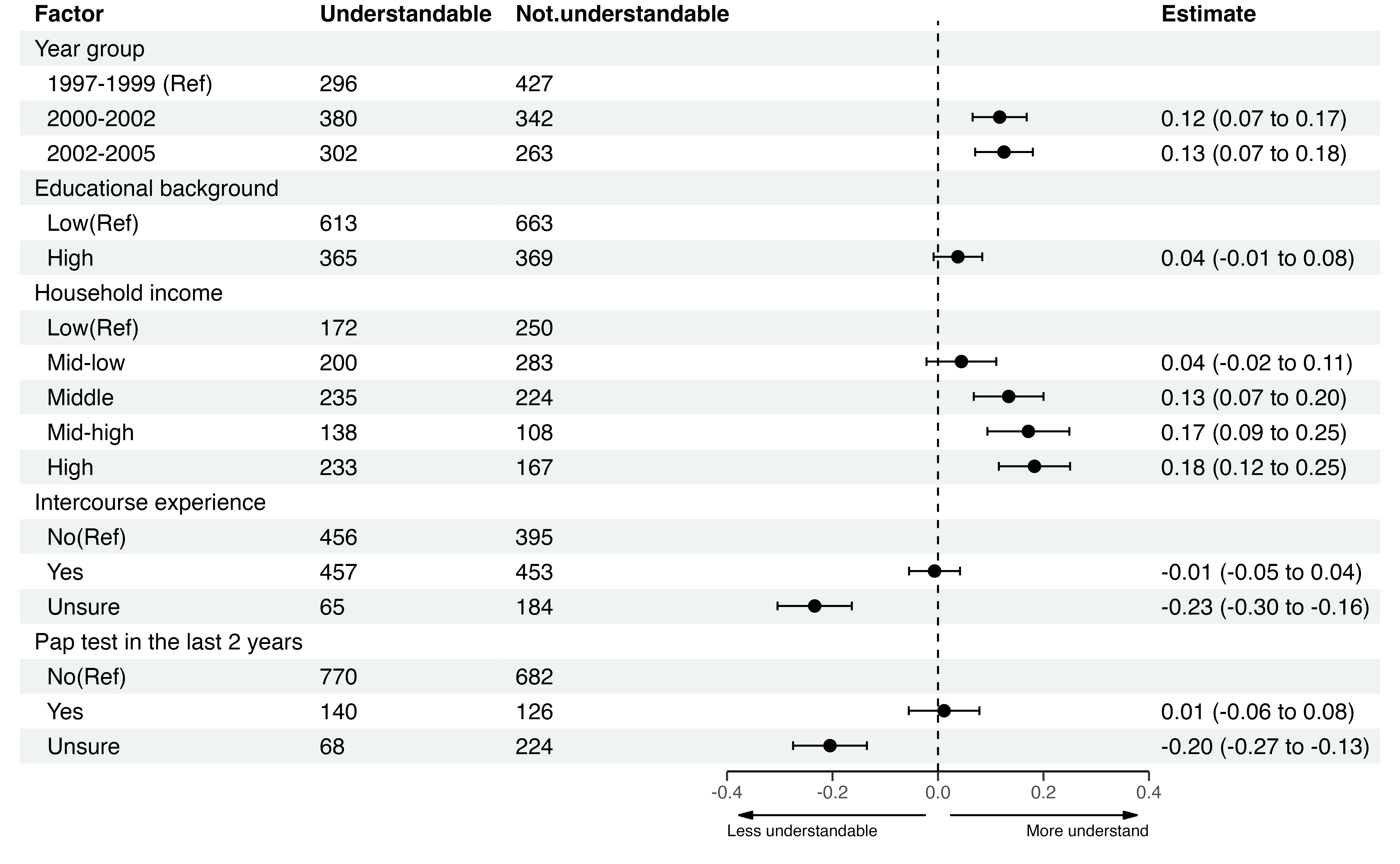


Figure S4

Reasons given by women for not planning to receive the HPV vaccine in the future.


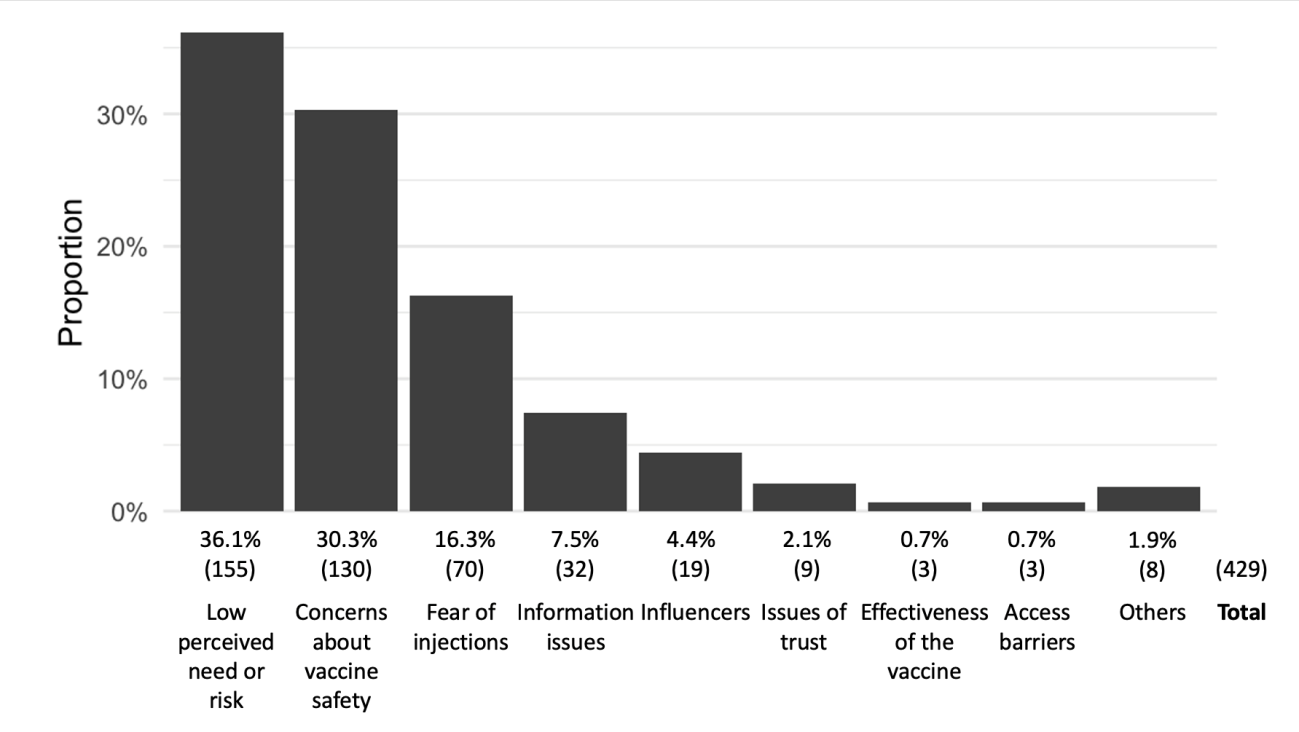

Supplement: Multimedia Appendix 1 [file jmir-v27-e67778-s001.docx]
